# Cytokine inducible SH2-containing protein: a versatile negative regulator of cytokine receptor signaling

**DOI:** 10.3389/fimmu.2026.1752876

**Published:** 2026-03-09

**Authors:** Wasan Naser, Alister C. Ward

**Affiliations:** 1 School of Medicine, Deakin University, Geelong, VIC, Australia; 2 Department of Biotechnology, College of Science, University of Baghdad, Baghdad, Iraq; 3 IMPACT, Deakin University, Geelong, VIC, Australia

**Keywords:** cytokine receptor signaling, erythropoietin, GM-CSF, IL-15, IL-2, IL-4, leptin, immunity

## Abstract

Cytokine inducible SH2-containing protein (CISH) was the founding member of the suppressor of cytokine signaling (SOCS) family of negative regulators. However, the subsequent elucidation of the physiological roles of CISH has been a slow process, reflecting its often subtle basal functions. Here we provide a narrative review of the literature highlighting the niche roles played by CISH principally in the control of cytokine signaling that impacts immune, blood and other cells. CISH regulates T cell production, polarization and activation through interleukin (IL)-2, IL-4 and the T cell receptor (TCR), natural killer (NK) cell production and activation via IL-15, generation and/or activation of neutrophil, dendritic cell (DC) and macrophage populations through granulocyte-macrophage colony-stimulating factor (GM-CSF), erythrocyte production via erythropoietin (EPO), and appetite control through leptin. Many of these roles are performed by CISH in concert with other SOCS proteins, providing additional complexity. CISH has also been identified in the etiology of several human diseases, particularly immune disorders, such as allergy and susceptibility to infectious disease, as well as a potential target to augment immunotherapy.

## 1 Introduction

**Figure 1:**
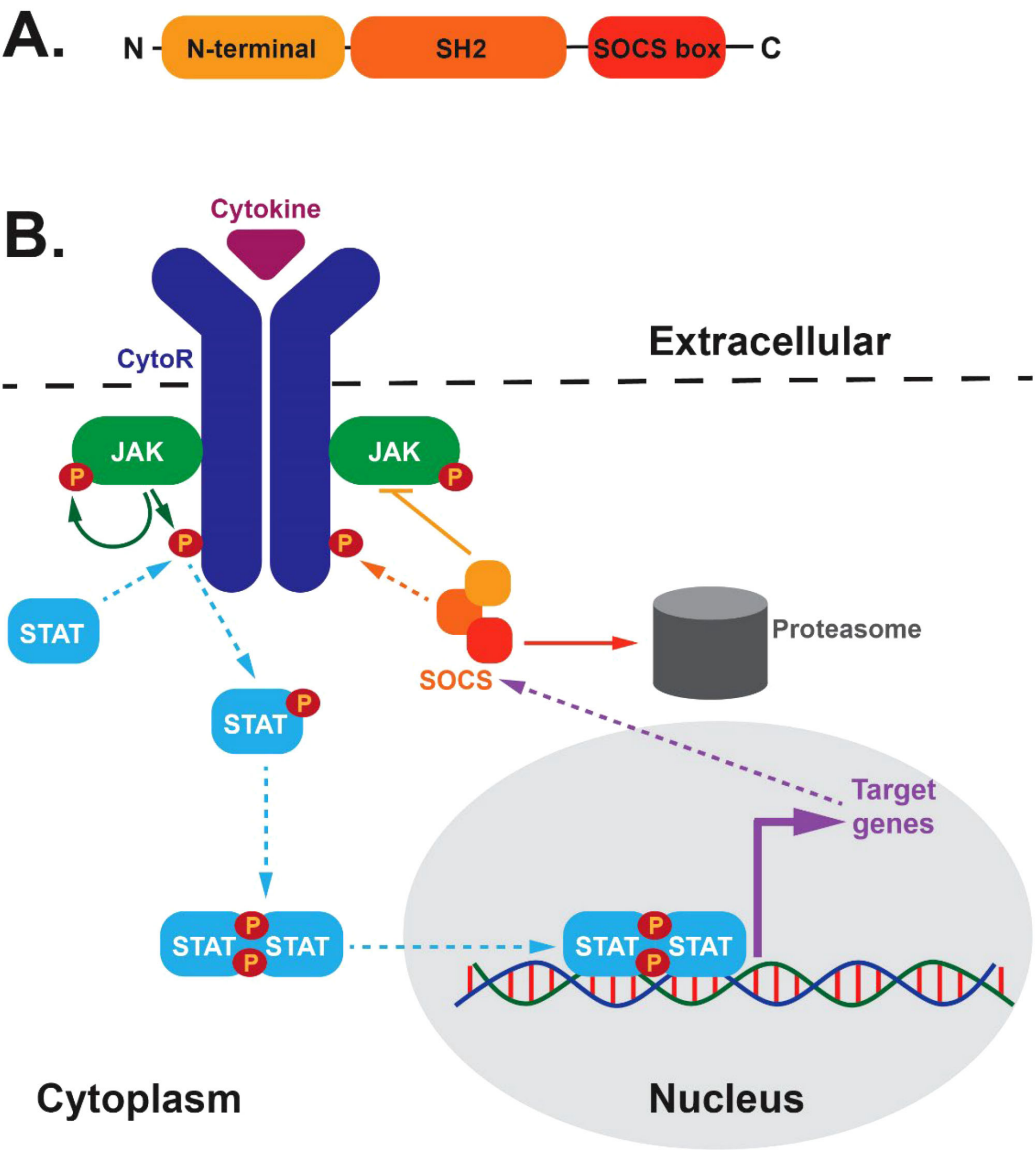
SOCS protein structure and function. **(A)** Domain structure of SOCS proteins. This includes a variable N-terminal domain and highly-conserved Src-homology 2 (SH2) and SOCS box domains. **(B)** Schematic representations of negative feedback regulation mediated by SOCS proteins. Cytokines (purple) bind extracellularly to their respective cytokine receptors (CytoR, dark blue) that facilitates activation of intracellular-associated Janus kinase (JAK, green) molecules that mediate tyrosine phosphorylation (P, brown) of the JAKs and CytoRs that allows docking of signaling molecules, including signal transducer and activator of transcription (STAT, light blue) proteins. These in turn become tyrosine-phosphorylated, dimerise and migrate to the nucleus to initiate transcription of target genes, including those encoding SOCS proteins. These are able to bind to the CytoR complex (as well as other signaling molecules) to negative regulate signaling through steric hindrance of signaling molecule docking, direct JAK inhibition or targeting of CytoR signaling components for proteasomal degradation.

Cytokines are critical mediators of diverse aspects of biology, most notably blood and immune cell development and function, but also extending to other aspects of homeostasis (1). A key aspect of cytokine function is their typically transient nature in part due to a range of negative regulators, such as members of the suppressor of cytokine signaling (SOCS) family (2). These proteins consist of a divergent N-terminal domain that is unstructured with no clear function, a central Src homology 2 (SH2) domain that binds phosphotyrosine motifs in target proteins and a so-called ‘SOCS box’ domain at the C-terminus that binds to E3 ubiquitin ligase complex components (**Figure 1A**). The SOCS family comprises SOCS1–7 and the alternatively-named cytokine inducible SH2-containing (CISH) protein, with SOCS1–3 and CISH involved in regulation of cytokine receptor signaling (3), reflecting their contemporaneous evolution with this signaling system (4).

SOCS-mediated negative feedback involves extracellular cytokines triggering activation of their cognate receptor complexes that initiates activation of intracellular signaling, particularly via the Janus kinase/Signal transducer and activator of transcription (JAK/STAT) pathway (**Figure 1B**). This results in the formation of tyrosine-phosphorylated STAT dimers that migrate to the nucleus to initiate transcription of target genes, including those encoding SOCS proteins that negatively regulate cytokine receptor signaling. This is achieved by binding to activated receptor complexes via their SH2 domain to directly block receptor docking sites and/or facilitate JAK inhibition and/or enable proteasomal degradation of receptor complex components mediated via their SOCS box (3).

A number of specific roles in cytokine receptor signaling have been elucidated. For example, SOCS1 in the regulation of signaling mediated by the receptors for interferon gamma (IFNγ), interleukin (IL)-12 and IL-2 family members (5), SOCS2 for the growth hormone (GH) receptor (6) and SOCS3 for IL-6 receptor (IL-6R) family members, including IL-6R, granulocyte colony-stimulating factor receptor (G-CSFR) and leukemia inhibitory factor receptor (LIFR) (7, 8). CISH, however, has been identified as a broader regulator across a range of cytokine and other receptors.

## 2 Discovery, characterization and conservation

*CISH*, also known as *CIS*, was discovered as an immediate-early gene induced following stimulation with the cytokines IL-2, IL-3, granulocyte-macrophage colony-stimulating factor (GM-CSF) and erythropoietin (EPO) (9). The authors further showed that enforced expression of this gene negatively regulated cytokine responses with the encoded protein able to directly bind tyrosine-phosphorylated receptor chains in the cytoplasm. CISH was subsequently found to be induced by a plethora of additional cytokines, such as IL-4, IL-6, IL-7, IL-15; G-CSF, thrombopoietin (TPO) and IFNγ (10–12). CISH also showed broad expression, being highly expressed in the liver, kidney and lung, and to a less extent in the heart, stomach, spleen and thymus (9, 10). CISH displayed good conservation across higher vertebrates, with multiple paralogues typically found in teleost fish, as exemplified by the *cish.a* and *cish.b* genes found in zebrafish and pufferfish (13).

A mutual regulatory network involving CISH and STAT5 has been identified that shows strong conservation between mammals and zebrafish. CISH is typically induced by cytokines that strongly activate STAT5 (9–12). Moreover, *Stat5a/b* knockout mice displayed reduced *Cish* expression (14, 15), while knockdown of zebrafish *stat5.1* reduced *cish.a* (but not *cish.b*) mRNA levels (13). Conversely, enforced expression of a constitutively-active form of zebrafish Stat5.1 lead to upregulation of *cish.a* (13). This key regulatory role for STAT5 in CISH gene expression is achieved by binding of STAT5 molecules in a tetrameric configuration via two pairs of tandem TTCNNNGAA motifs (16), which are conserved in the promoters of mammalian *CISH* and zebrafish *cish.a* genes, with one pair found in the zebrafish *cish.b* promoter (13). Alternatively, STAT5 activation was suppressed in transgenic Tg(*Actb*::CISH) mice expressing CISH from the constitutive and ubiquitous β-actin promoter (17) and elevated in zebrafish embryos in which *cish.a* was ablated (13) as well as in *Cish* knockout mice (12, 18). However, CISH can also be induced by other STAT proteins including STAT6 activated by IL-4R (19) and regulate pathways involving STAT3 and STAT6 (19, 22, 57).

## 3 Roles in immune cells

### 3.1 T cells

**Figure 2:**
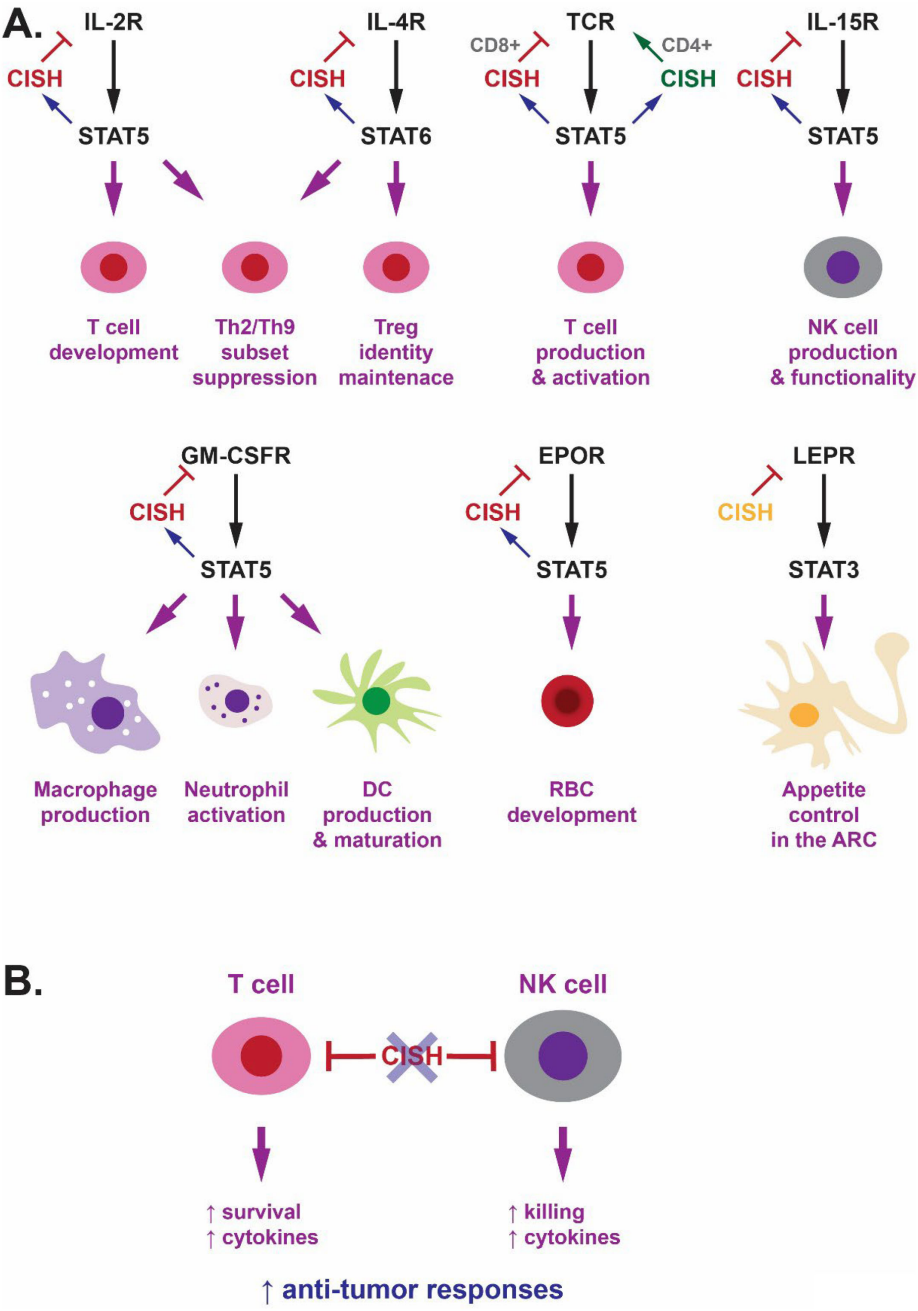
Key roles of CISH in the regulation of signaling from cytokine and other Receptors and its targeting to augment cancer immunotherapy. **(A)** Schematic representation of the specific signaling pathways regulated by CISH and their respective cellular impacts. CISH is shown in red where it is clearly part of a negative feedback loop, with alternative regulatory modes shown in other colors. **(B)** Diagram of CISH as an immune checkpoint inhibitor and the therapeutic benefits of its ablation on anti-tumor responses.

CISH has been shown to play a variety of roles impacting T cell development, homeostasis and function, the majority of which involve regulation of cytokine signaling (**Figure 2A**). Early studies of transgenic Tg(*Actb*::CISH) mice identified a reduced ratio of T helper (Th)1 to Th2 cells and decreased γδ T cells, which correlated with dampened IL-2R mediated T cell proliferation and other responses (17). IL-2 has been demonstrated to strongly induce CISH expression in a STAT5-dependent manner (9, 20), with CISH able to associate with the IL-2Rβ chain to inhibit IL-2R signaling (21), suggesting a negative feedback regulatory function in this context. This may also explain the increased progenitor and mature T cells following *cish.a* ablation in embryonic zebrafish, where IL-2Rβ is conserved (13).

Further insights have been obtained from the generation and analysis of *Cish* knockout mice. Global *Cish* knockout mice developed spontaneous allergic pulmonary disease with aging, exhibiting hyperplasia of alveolar epithelial cells and muscle cells in lung and excessive eosinophils in broncho-alveolar lavage (19). They also showed enhanced susceptibility to experimental allergic asthma, which could be recapitulated in CD4+ T cell – but not regulatory T (Treg) cell – conditional knockouts. The allergic phenotypes resulted from enhanced signaling via IL-4R/STAT6 and IL-2R/STAT5 in CD4+ T cells, facilitating increased generation of Th2 and Th9 subsets leading to excessive production of IL-4 and IL-9, respectively (19). IL-4 was found to be the major inducer of CISH in CD4+ T cells at least partially via STAT6 (19), suggesting a classic negative feedback loop involving CISH directly impacts effector cells. However, on a different genetic background, Treg-conditional *Cish* knockout mice displayed spontaneous eosinophilic airway inflammation and exacerbated experimental asthma (22). This correlated with a loss of Treg identity and suppressive functions and an enhancement of effector programs, including increased production of the signature Th2 cytokines IL-4 and IL-5, leading to unrestrained autocrine IL-4R/STAT6 signaling (22). This alternative indirect mechanism could clearly synergize with the direct role in effector cells. Interestingly, the development of allergic disorders in infants was associated with elevated levels of an effector memory Th2 population displaying elevated levels of CISH, although whether this was a cause or consequence was unable to be determined (23). Another recent study has implicated CISH in chronic inflammation associated with aging. Activated T cells in older adults were shown to have elevated levels of CISH, which mediated proteasomal degradation of the ATPV1A proton pump critical for lysosome function leading to release of mitochondrial DNA that triggered inflammation. Silencing of CISH in CD4+ T cells restored lysosomal activity, reducing mtDNA release and pro-inflammatory cytokines (24).

CISH has also been implicated in the regulation of T cell receptor (TCR) signaling, with CISH found to be induced by TCR stimulation in both CD4+ (25) and CD8+ (26) T cells. However, the impacts were cell subset dependent. Thus, CD8+ cells derived from *Cish* knockout showed enhanced expansion, functionality and anti-tumor activity mediated by increased production of cytokines such as IFNγ, tumor necrosis factor (TNF)α or β and IL-2, which correlated with increased TCR signaling. The suppressive role of CISH on TCR signaling was attributed to a direct interaction with the TCR signaling intermediate phospholipase C (PLC)γ1 that it targeted for proteasomal degradation (26). In CD4+ T cells, however, enforced expression of CISH resulted in elevated TCR-dependent proliferation, cytokine production and superantigen-induced T cell activation as well as prolonged survival. This effect instead resulted from interaction of CISH with protein kinase C (PKC)θ enabling increased activation of mitogen-activated protein kinases (MAPKs) downstream of the TCR (25).

### 3.2 NK cells

CISH has also been identified as an important regulator in natural killer (NK) cells (**Figure 2A**). *Cish* knockout mice showed normal basal levels of NK cells, however enhanced terminal differentiation was observed leading to a higher proportion of mature NK cells, especially in the spleen. There was also increased cell cycling but also elevated turnover that together resulted in the overall NK cell frequency being within the normal range (27). NK cells from *Cish* knockout mice showed enhanced sensitivity to IL-15 resulting in increased proliferation, survival and effector functions, including IFNγ production and tumor cytotoxicity. *Cish* was found to be rapidly induced by IL-15 in NK cells, with *Cish* ablation resulting in enhanced JAK-STAT signaling concomitant with increased levels of the IL-15R component IL-2Rβ, along with the associated JAK1 and JAK3. This was likely mediated by direct interaction with JAK1 and IL-2Rβ, facilitating both JAK inhibition and proteasomal targeting of receptor signaling components (12). Collectively this indicates CISH is a negative regulator of IL-15R. Tg(*Actb*::CISH) mice displayed drastically reduced number of NK cells (17), which potentially could be explained at least partially via this mechanism.

*Cish* knockout mice were resistant to melanoma, prostate, breast cancer and lung metastases (12, 28), which was dependent on NK cells but not CD4+ T-cells (28). This augmented anti-tumor functionality correlated with a lower NK cell activation threshold, suggesting that CISH is an important intrinsic checkpoint in NK-cell immunity (12). *Cish* deficient NK cells were also found to be hyporesponsive to tumor growth factor (TGF)β, with deletion of the *Tgfbr2* gene leading to similar robust resistance to tumor development as *Cish* knockout mice (29). Tumor resistance in *Cish* knockout mice was also dependent on IFNγ (28). Together this suggests dysregulation of a broad cytokine network is likely involved.

*Cish* knockout mice also displayed resistance to *de novo* tumor formation in a carcinoma-induced sarcoma model, but progression of hematological tumors was normal (28). In contrast, another study showed that deletion of *CISH* in human induced pluripotent stem cells (iPSC)-derived NK cells resulted in enhanced inhibition of tumor progression in a leukemia xenograft model (30). The reason for this difference remains unclear. These NK cells also showed increased *in vivo* persistence, which correlated with improved metabolic fitness, including increased basal glycolysis (30). This contrasted with NK cells from *Cish* knockout mice that showed decreased expression of metabolic genes, including those associated with glycolysis (27).

### 3.3 Myeloid cells

Adult *Cish* knockout mice exhibited elevated basal neutrophil numbers in the blood, in concert with increased frequency of neutrophils (and total myeloid cells) in the bone marrow and spleen (31). Ablation of the zebrafish *cish.a* paralog similarly resulted in increased number of neutrophils during both primitive and early definitive hematopoiesis (13). Together this is consistent with a conserved non-redundant role for CISH in the regulation of basal neutrophil production. The *Cish* knockout mice additionally showed an increase in basal myeloid and total dendritic cells (DCs) in the bone marrow (31). It remains to be elucidated the mechanism by which CISH exerts these basal effects.

Ablation of *Cish* also significantly impacted GM-CSF-mediated myelopoiesis (**Figure 2A**). There was an increased frequency of neutrophils and total myeloid cells in the bone marrow, and elevated colony-forming unit (CFU)-G frequency in the spleen (31). In an alternate *Cish* knockout model, neutrophils showed prolonged survival and enhanced expression of chemokines and other pro-inflammatory molecules, with the mice displaying exacerbated neutrophil-dependent joint and CNS inflammation. *Cish* knockout mice also showed exaggerated GM-CSF driven responses in the monocyte/macrophage lineage. This included accelerated GM-CSF-mediated differentiation into CD11c+ macrophages and higher chemokine expression (18), but also perturbation of homeostatic GM-CSF-mediated alveolar macrophage (AM) production leading to the generation of foamy AMs and accumulation of pulmonary surfactant (32), as well as skewing toward immunosuppressive M2-like macrophages (33). GM-CSF induced sustained levels of *Cish* in mouse bone marrow cells, while *Cish* knockout mice exhibited prolonged GM-CSF-driven signaling, especially of STAT5 and MAPK members (18, 32). This correlated with increased levels of the GM-CSFR β-chain due to altered recycling, with a potential CISH binding site identified at Y822 of the mouse protein (18). These results are consistent with an *ex vivo* study that identified strong induction of *Cish* expression during GM-CSF-mediated DC development from mouse bone marrow cells that matched the kinetics of STAT5 activation and was ablated by a STAT5 inhibitor (34). Knockdown of *Cish* in these cells resulted in increased STAT5 activation concomitant with elevated DC proliferation but decreased expression of MHC class I and pro-inflammatory cytokines. It also inhibited the development of type I DCs essential for CD8+ activation to become effective stimulators of cytotoxic T lymphocytes as part of anti-tumor immunity (34). Together this suggests CISH acts as both a negative feedback and homeostatic regulator of GM-CSF via the GM-CSFR β-chain impacting multiple aspects of myeloid cell development.

*Cish* knockout mouse bone marrow cells also generated greater myeloid progenitors in response to IL-3 and eosinophils in response to IL-5, the receptors for which share the same or equivalent β-chain as GM-CSFR (18). Indeed, CISH has been shown to be induced by IL-3 and form a complex with tyrosine phosphorylated IL-3 β-chain and block IL-3 mediated STAT5 phosphorylation (9, 20). This suggests the potential for a similar negative feedback loop in these instances. In contrast, *Cish* ablation had little impact on G-CSF-mediated myelopoiesis, apart from an increased frequency of CFU-G and CFU-M in the spleen, with unaltered levels and kinetics of G-CSF-induced STAT5 activation in bone marrow cells (31). This was consistent with another report showing CISH was unable to inhibit G-CSF-mediated STAT activation (35), despite *in vitro* studies showing that G-CSF can induce CISH, with CISH able to bind phosphopeptides corresponding to those found on the G-CSFR (36).

### 3.4 Other immune-related cells

CISH has also been shown to influence mucosal immunity in other ways, including constraining a regulatory circuit involving type 2 innate lymphoid (ILC2) cells and Tuft cells to set immune and epithelial tone. Thus, CISH was found to be highly expressed in ILC2 cells, while both global and ILC2-specific *Cish* knockout showed an increase in ILC2 expansion and activation, resulting in an increase in basal and inflammation-induced Tuft cells (37). In contrast, other research with a global *Cish* knockout mice showed normal numbers of lung-resident ILC2 cells (12), suggesting potential tissue-specificity in this role.

### 3.5 Infectious disease susceptibility

Human studies have implicated CISH in infectious disease susceptibility, which is perhaps not surprising given the multiple regulatory roles identified in immune cells. Five single nucleotide polymorphisms (SNPs) in human *CISH* were associated with significant enhanced susceptibility to malaria, bacteremia and tuberculosis (TB), although the overall enhancement was only around 20% with most of the significance due to SNP rs414171 (located in the promoter region at -292), which correlated with blunted IL-2-mediated CISH expression (38). Associations between CISH SNPs and susceptibility to viral infections have subsequently been reported (39).

The rs414171T variant was associated with increased susceptibility to bacteremia (38), as well as to sepsis and multiple organ disruption syndrome (MODS) in trauma patients, with these patients showing reduced lipopolysaccharide (LPS)-mediated induction of CISH and tumor necrosis factor (TNF)α (40). Another SNP, rs143356980 was associated with increased risk of death in sepsis patients (41). Moreover, global and ILC2-specific *Cish* knockouts displayed compromised control of the model bacteria *Salmonella typhimurium*, which contrasted to accelerated clearance observed for the model helminth *Nippostrongylus brasiliensis* (37). Homozygous rs414171 TT variants conferred significantly increased susceptibility to hepatitis B virus (HBV) (39), with another study in a Vietnamese population suggesting a gene dosage effect (39). Moreover, rs414171AA homozygosity was higher in patients with resolved compared to permissive HBV (42).

Association of the rs414171 T allele was also significantly associated with malarial susceptibility across multiple cohorts (38). However, *Cish* knockout mice were not significantly different in their responses to malaria infection, including similar kinetics, parasite load, weight loss and cytokine responses. In fact, intriguingly, *Cish* knockouts showed better maintenance of circulating red blood cell parameters as well as bone marrow and spleen erythropoiesis during infection, despite these being impacted in uninfected knockouts (43).

Significant association between CISH SNPs and TB was observed in some cohorts but not others (38). In addition, the direction of association was variable even within the same ethnic cohort. For example, one study showed that rs414171 TT homozygosity and rs809451 GC heterozygosity were associated with increased risk of TB, associated with reduced CISH and increased IL-10 and IL-12p40 (44), whereas another study showed rs414171 AA homozygosity mediated increased TB risk (45). There was also a case report describing a patient with recurrent TB who possessed multiple *CISH* gene mutations (46). In an experimental infection model, *Cish* knockout mice displayed a transient increase in *M. tuberculosis* load in the spleen and lung with levels of TNF reduced, and transplantation studies suggesting innate immune involvement. However, the mice showed normal neutrophil recruitment, similar mycobacterial uptake and growth in macrophages, while long-term outcomes were normal (47). CISH was separately found to be required for *M. tuberculosis* mediated Treg expansion (48), indicating a role in adaptive immunity. In contrast, another study demonstrated that inhibition of CISH reduced replication of *M. tuberculosis.* Infection of macrophages with *M. tuberculosis* caused GM-CSF secretion, leading to enhanced STAT5-mediated CISH induction, which was able to target ATPV1A, thereby preventing phagosome acidification (49, 50).

## 4 Roles in non-immune cells

### 4.1 Red blood cells

Adult *Cish* knockout mice showed disrupted basal bone marrow erythropoiesis, with decreased CFU-erythroid and blast-forming unit (BFU)-E precursors, elevated pro-, basophilic, polychromic and orthochromic erythroblasts, but reduced reticulocytes. There was evidence of compensation in the spleen, where CFU-E, BFU-E and pro-erythroblast populations were elevated (51). This collectively correlated with a small but significant reduction in hemoglobin and hematocrit in concert with increased mean red cell volume, consistent with mild macrocytic anemia. Responses to EPO injection were blunted and delayed in the bone marrow, in contrast to higher albeit more transient responses in the spleen (51). *Cish* knockout mice also displayed slightly elevated fetal liver cellularity with a significant increase in the relative number of TER199+ erythroid cells, which correlated with strong *Cish* expression in this organ that peaked at embryonic day 12.5 (E12.5) (51). This timing was broadly coincident with the initiation of EPOR-mediated definitive erythropoiesis (52). Overexpression of CISH in mouse fetal liver cells has been shown to impair proliferation, but not differentiation, and increase apoptosis of erythroid progenitors stimulated with EPO (53). The zebrafish *cish.a* gene was expressed in embryonic caudal hematopoietic tissue that shows functional analogy with mammalian fetal liver, while knockdown of zebrafish *cish.a* (but not *cish.b*) resulted in a transient increase in erythroid precursors and mature erythroid cells during embryonic erythropoiesis (13), which is also an EPOR-dependent process (54). CISH was previously found to be induced *in vitro* by EPO via the JAK-STAT5 pathway (9), with enforced expression of CISH able to reduce EPO-mediated STAT5 activation (20). CISH was able to bind to EPOR via its SH2 domain at Y401 and to a lesser extent Y344 (53, 55), inhibiting signaling by direct competition with STAT5 that binds the same sites (55), in addition to proteasomal-mediated degradation of the receptor (56). This collectively points to a conserved role for CISH as a negative feedback regulator of EPOR/STAT5-dependent erythropoiesis (**Figure 2A**), with different effects depending on the stage of red blood cell development.

### 4.2 Arcuate nucleus

Male *Cish* knockout mice displayed significantly decreased adiposity, particularly impacting mesenteric fat (57), which was consistent with human studies that identified an association between increased CISH expression and obesity (58). The *Cish* knockout mice were also resistant to high-fat diet induced obesity, hepatosteatosis and insulin resistance. This was accompanied by reduced food intake, but unaltered basal metabolism, energy expenditure, physical activity and microbiota (57). *Cish* was found to be expressed in the arcuate nucleus (ARC), as described in a previous study (59), with deeper analysis identifying co-expression with the *Lepr* gene in neurons expressing the orexigenic Agouti-related peptide (*Agrp*) and some expressing the anorexigenic proopiomelanocortin (*Pomc*). *Cish* knockout mice showed reduced basal expression of the *Agrp* gene and increased leptin-mediated expression of the *Pomc* gene and suppression of food intake (57). Other *in vitro* studies have identified direct interaction between CISH and Y985 and Y1077 of the LEPR (60). Meanwhile mice carrying a Y785F mutation in LEPR displayed leptin hypersensitivity only in males (61). Together this suggests that CISH acts as a direct chronic regulator of LEPR-mediated appetite control via STAT3 (**Figure 2A**), rather than a negative feedback regulator, since leptin did not significantly impact *Cish* expression (57). However, another study found reduced *Cish* expression in *Lepr* deficient mice (62), suggesting some relationship between LEPR signaling and CISH expression. Ablation of *Gmcsfr* failed to impact these phenotypes, ruling out a role for CISH in GM-CSFR-mediated appetite regulation (57).

### 4.3 Other roles

In addition to the various roles for CISH in regulating immune cell-mediated inflammation, other research suggests it may also influence inflammation via alternative mechanisms. For example, IL-13 was demonstrated to strongly induce CISH via STAT6 in human lung fibroblasts, with CISH able to negatively regulate IL-13-mediated induction of the chemokine *CCL26*, which acts potently on eosinophils (63). Furthermore, CISH has been shown to be elevated in the colons of aged mice and older ulcerative colitis patients. Intestinal epithelial cell-specific *Cish* knockout prevented experimentally-induced colitis in older mice, which was associated with suppression of oxidative stress and concomitant proinflammatory responses (64).

Other development roles have been suggested for CISH. Tg(*Actb*::CISH) mice showed reduced body weight, concomitant with reduced GH-mediated STAT5 activation and expression of MUP (17). The Tg(*Actb*::CISH) mice also showed impaired mammary gland development and failure to lactate, including reduced expression of whey acidic protein, with CISH expression able to inhibit PRL-mediated STAT5 activation (17). Some of these roles are consistent with relevant *in vitro* studies on the effects of CISH on signaling from the respective receptors (65, 66), which was similarly observed in Stat5b knockout mice (67). No analyses of GH and PRL responses in CISH knockout mice have been published information, which might shine further light onto its physiological role.

There is also support for CISH having a role in the regulation of hepatic gluconeogenesis. Glucagon levels were elevated in *Cish* knockout mice, indicating an impact on pancreatic β-cell function or on glucagon signaling in responsive tissues including liver (57). However, these mice had no defects in insulin production or secretion (57), which was consistent with mice harboring a pancreas-specific *Cish* knockout allele that showed normal β-cell proliferation, insulin production and glucose tolerance during pregnancy (68). In contrast, overexpression of CISH in mouse hepatocytes *ex vivo* or *in vivo* resulted in decreased levels of key gluconeogenic enzymes via the CREB transcription factor, resulting in reduced glucose production and improved glucose tolerance, while CISH knockdown had the opposite effect (62).

## 5 Interplay with other SOCS proteins

CISH does not act in isolation. Instead there is considerable evidence that it often works in concert with other SOCS family members. This can reflect common and/or parallel pathways, but also distinct and antagonistic functions. This creates the potential for redundancy and/or compensation but ultimately underpins exquisite multi-level control. For example, SOCS3 has also been shown to regulate EPO-mediated fetal erythropoiesis (69), as part of a negative feedback loop that similarly blocks STAT5 docking (56). SOCS3 also regulates LEPR-mediated appetite control, although this is part of clear negative feedback loop (70), while SOCS2 appears able to block CISH-mediated control of LEPR signaling (60). Like CISH, SOCS2 regulates Treg stability by regulating IL-4/STAT6 signaling (71), whereas SOCS1 achieves this by regulating STAT1 and STAT3 (72). It has also been demonstrated that like CISH, SOCS1 can negatively regulate GM-CSFR-mediated responses, including DC production and activation (73, 74), but this most likely represents cross-regulation that only occurs at high levels of SOCS1 (74). In NK biology, SOCS2 regulates basal NK cell production (75), rather than the IL-15R-mediated NK cell expansion and activation controlled by CISH, while NKT activation is controlled by SOCS1 (76). SOCS3 negatively regulates neutrophil production mediated by G-CSF (77) but not that mediated by GM-CSFR. Multiple SOCS proteins also act in the context of glucose metabolism (78–81).

## 6 Therapeutic targeting

CISH is an intrinsic checkpoint inhibitor that dampens anti-tumor immune functions in T cells (26), NK cells (12) and DC cells (34). This has initiated studies targeting CISH for immunotherapy applications (**Figure 2B**). For example, ablation of CISH in primary allogeneic NK cells resulted in increased effector pathways, such as those mediated by IFNγ and TNFα, enhanced NK-mediated killing of glioblastoma *in vitro* and prolonged survival of mice harboring brain cancer xenograft (82). Ablation of CISH in chimeric antigen receptor (CAR)-T cells using genome editing approaches resulted in increased survival and cytokine secretion of these cells that was associated with enhanced anti-tumor activity. This was mediated by reduced PD-1 expression caused by increased levels of the ubiquitinating protein FBX038 in the absence of CISH (83). Importantly, the first in-human phase I trial has been completed utilizing tumor-infiltrating lymphocytes in which CISH was ablated with genome editing tools, that demonstrated safety as well as potential for anti-tumor response (84). Importantly, *Cish* knockout mice have been shown to remain sensitive to anti-PD-1/CTLA-4 checkpoint inhibitors, as well as both IFNα/β and IL-2, while *Cish* ablation synergized with combined BRAF and MEK inhibition in a mouse metastatic melanoma model (28). This suggests that combinatorial approaches involving CISH genome editing in concert with traditional therapies will be possible.

## 7 Conclusions

CISH represents a versatile regulator of cytokine signaling in health, the disruption of which is associated with immune and infectious diseases. The majority of the impacts of CISH basally are quite subtle, with its regulatory actions instead becoming more visible in response to physiological stresses. Thus, the basal impact of CISH ablation on specific lymphoid cell populations is rather limited (12, 26). Indeed, the only significant pathological consequence reported (airway inflammation) was limited to aging *Cish* knockout mice (19). However, the effects of CISH ablation were strongly exacerbated in a setting of experimental allergic asthma, where the impacts on IL-4R signaling became very apparent (19). Similar, it is in the context of tumor challenge that the effects on TCR (26) and IL-15R (12) signaling became most overt, being manifest by significantly enhanced anti-tumor responses. Similarly, for red blood cells and neutrophils, ‘emergency’ conditions – as simulated by EPO and GM-CSF injections, respectively – are necessary to reveal critical roles for CISH (31, 51). This also extends to appetite control and adiposity, with the basal impacts of CISH being significantly exaggerated when mice were placed on a high-fat diet (57). CISH performs these functions as part of a sophisticated regulatory network with other SOCS proteins, which requires further investigation for full understanding. Finally, targeting of CISH represents an exciting approach in the context of anti-tumor immunity that will likely have additional clinical applications in the future.
